# A Review of the Current Literature on Cerebral Aneurysms

**DOI:** 10.7759/cureus.80223

**Published:** 2025-03-07

**Authors:** Tyler E Rice-Canetto, Arisa Ueno, Eric Whitney, Louis Reier, Rebecca Houston, Javed Siddiqi

**Affiliations:** 1 Neurosurgery, Arrowhead Regional Medical Center, Colton, USA; 2 Neurosurgery, California University of Science and Medicine, Colton, USA; 3 Neurosurgery, Riverside University Health System Medical Center, Moreno Valley, USA; 4 Neurosurgery, Desert Regional Medical Center, Palm Springs, USA

**Keywords:** aneurysm, cerebral aneurysm, cerebral berry aneurysm, cerebral blister aneurysm, cerebral fusiform aneurysm, cerebral marantic aneurysm, cerebral mycotic aneurysm, cerebral saccular aneurysm, dissecting cerebral aneurysm

## Abstract

Cerebral aneurysms may be classified by type, size, location, and ruptured versus unruptured status. Each type of aneurysm has associated epidemiology, pathophysiology, clinical features, diagnostic protocols, management strategies, and prognoses. Optimization of clinical outcomes requires an in-depth understanding of each of these factors for a given aneurysm type, in combination with a tailored approach for each patient. This review will encompass six different types of intracranial aneurysms: saccular, fusiform, dissecting, mycotic, marantic and blister aneurysms. The clinical presentation of each aneurysm type varies based on its underlying etiology as well as its rupture status, and may range from asymptomatic to hemorrhage. Diagnostic tools generally include imaging with some combination of computerized tomography, magnetic resonance imaging, and angiography, in addition to investigations appropriate for the underlying cause. Management consists of a combination of medical and possible surgical interventions tailored to the unique characteristics of each aneurysm and patient. Lastly, prognosis varies widely and is dependent upon a number of factors, including but not limited to aneurysm type, rupture status, intervention modality, development of complications, and patient demographics and comorbidities. While abundant literature on the more common cerebral aneurysmal types of saccular, fusiform, and mycotic exists, little is available on the remaining three. Additionally, the six subtypes have rarely been summarized together in one review paper with succinct differentiations and comparisons. Having an in-depth understanding of each cerebral aneurysm type, their similarities and differences, and the interplay of patient specific factors will help guide medical and surgical intervention and allow providers to more accurately disseminate patient prognosis. We aim to summarize the current literature on cerebral aneurysms in order to inform clinical decision making and optimize patient outcomes.

## Introduction and background

Intracranial aneurysms develop from a weakened segment of a cerebral vessel wall resulting in abnormal focal dilation [1]. Unruptured intracranial aneurysms are present in an estimated 3.6% to 6% of the general population, with a rupture rate of approximately 0.01% annually [2,3]. Individuals who have two or more relatives with brain aneurysms have a higher incidence, which is between 8% and 9% [2]. Cerebral aneurysms are most common between 35 and 60 years of age and are more prevalent in women than in men by a 3:2 ratio [4]. When compared to their Caucasian counterparts, African American and Hispanic populations are at two times the risk for aneurysm development [4]. There are numerous risk factors for cerebral aneurysms, including inherited conditions and lifestyle or other factors. These include but are not limited to autosomal dominant polycystic kidney disease, connective tissue disorders, smoking, drug use, head trauma, female gender, and age greater than 50 years [2]. 

While saccular aneurysms account for 90% of all cerebral aneurysms, there are other subtypes that providers should be familiar with [2]. These include fusiform aneurysms, dissecting aneurysms, mycotic aneurysms, marantic aneurysms, and blister aneurysms. Each has a distinct etiology, clinical presentation, diagnostic work-up, management, and prognosis. Additionally, each aneurysm type has unique characteristics such as size, location, and morphology that dictate appropriate intervention measures and help predict anticipated outcomes and complications. The aim of this paper is to summarize the current literature on each of these six types of intracranial aneurysms to help inform clinical decision making. We will provide information on the epidemiology, pathophysiology, clinical features, diagnosis, management, and prognosis for each aneurysm type as it pertains to clinical management.

## Review

Methods

This review summarizes the current literature on various intracranial aneurysm types including saccular, fusiform, mycotic, marantic, blister, and dissecting aneurysms with a focus on their epidemiology, pathophysiology, clinical features, imaging characteristics, management, and prognosis. A literature search was conducted using PubMed and Medline databases to identify relevant studies published. We selected filters for English language, human subjects, and a publication type of "article in press" or "review." Search terms included “saccular aneurysm”, “fusiform aneurysm”, “mycotic aneurysm”, “marantic aneurysm”, “non-bacterial thrombotic aneurysm”, blister aneurysm”, and “dissecting aneurysm”. In addition, references from relevant literature were manually reviewed to select for appropriate studies. They were considered for inclusion in the literature review if their primary focus was on cerebral aneurysmal factors such as general characteristics, diagnostic modalities, and therapeutic strategies for a given aneurysm type. Each pre-selected paper underwent a screening process by our authors for appropriate credibility in addition to quality of research methods and data analysis/presentation. Relevant data such as pathophysiology, presentation, imaging findings, risk of rupture, and treatment methods were extracted and synthesized systematically. Our exclusion criteria were any studies not focusing on these variables of interest, those restricted to a particular age group, animal and laboratory studies, novel diagnostics or therapeutics, clinical trials, publications requiring payment for access, and non-scientific publication types such as editorials and opinion columns. As with any literature review, there is some degree of possible selection bias, but our authors minimized this through predetermined inclusion and exclusion criteria as well as crosschecking of final paper selections. 

Saccular (berry) aneurysms

Definition and Epidemiology

Saccular aneurysms, also referred to as berry aneurysms, are the most common type of cerebral aneurysm, accounting for approximately 90% of all intracranial aneurysms [1]. They are present in 3% to 5% of the population, with a mean age of 50 at time of diagnosis and a predominance in females. Between 0.7% and 1.9% of saccular aneurysms rupture, leading to subarachnoid hemorrhage (SAH) [5].

Nearly 90% of saccular aneurysms are located in the Circle of Willis, with the remaining 10% predominately found at vessel bifurcation points [1,5]. Common sites within the anterior circulation include the junction points between the anterior communicating artery (AComm) and anterior cerebral artery (ACA), the posterior communicating artery (PComm) and internal carotid artery, and the bifurcation of the middle cerebral artery (MCA) [5]. AComm aneurysms have the highest chance of rupturing, accounting for approximately 35% of ruptured saccular aneurysms. They are also typically more complex given that they have a dual inflow and two to three outflow branches [6]. Within the posterior circulation the most common locations are at the top of the basilar artery, the junction between the basilar artery and superior cerebellar artery (SCA) or anterior inferior cerebellar artery (AICA), and the junction between the vertebral artery and posterior inferior cerebellar artery (PICA) [5].

Pathophysiology

Saccular aneurysms are generally the result of a weakened segment in the blood vessel wall, and typically form at vessel branch points where the impact of hemodynamic stress is high [5]. A more specific theory for the pathophysiology of berry aneurysm formation is the Coanda Effect, which describes the propensity of blood to adhere to a vessel wall opposite from a convex structure such as a cholesterol plaque [1]. The resulting decrease in flow resistance at the affected vessel wall creates a concavity. As blood is continually directed through the vessel concavity, a saccular aneurysm ensues [1]. Repetitive trauma or shearing forces at the vessel’s weak point causes aneurysmal enlargement [5]. 

There are various genetic and environmental factors that may increase one’s propensity for developing a saccular aneurysm. Genetic factors include various connective tissue disorders that weaken blood vessel walls, polycystic kidney disease, presence of arteriovenous malformations, hypercholesterolemia, and a history of aneurysm in two or more first degree family members [5]. Environmental risk factors include hypertension, smoking, stimulant drug use, and heavy alcohol consumption [5]. 

Clinical Features 

The clinical presentation of saccular aneurysms varies depending on rupture status, ranging from asymptomatic to SAH [5]. For unruptured aneurysms, most are diagnosed incidentally, but approximately 5% to 6% of patients are diagnosed based on mass effect symptomatology [7]. This most frequently includes oculomotor nerve palsy from aneurysms located in the PComm or visual disturbances secondary to optic nerve compression by carotid paraclinoid saccular aneurysms [7]. Patients with ruptured aneurysms will present with signs and symptoms of SAH common to all aneurysm types. This may include a myriad of clinical signs including a characteristic thunderclap headache that the patient may describe as the “worst headache of my life”, altered level of consciousness, focal or general seizures (present in up to 25% of SAH), meningeal signs such as neck pain or stiffness, focal neurological deficits, or visual impairment [5]. There are numerous cited risk factors that increase the risk of saccular aneurysm rupture. These include prior aneurysm rupture, smoking, hypertension, large size, aneurysms located in the posterior circulation, high rate of growth during the surveillance period, family history of aneurysms, and aneurysms which are irregularly shaped [5]. 

Diagnosis and Imaging

Diagnosis of saccular aneurysms is typically made via a sequence of cerebral imaging techniques (dependent on clinical presentation). Non-contrast head computerized tomography (NCCT) will detect SAH and will often prompt vessel imaging. Computerized tomography angiography (CTA) and magnetic resonance angiography (MRA) are both highly sensitive non-invasive tests for detecting brain aneurysms. CTA is faster to obtain, more readily available, and more cost effective. MRA is useful in patients who have kidney disease or any other contraindication to iodine-based contrast (which is used for CTA but not MRA). The gold standard diagnostic modality is a digital subtraction angiography (DSA) to evaluate exact aneurysm size, location, and morphology [2,5]. DSA is more invasive and time consuming but superior to the other vascular imaging studies due to its ability to evaluate cerebral blood flow in real time. In rare cases where patients have a negative NCCT but SAH is highly suspected, lumbar puncture may be considered to check for the presence of xanthochromia, a yellow tint to the cerebrospinal fluid as a result of the enzymatic breakdown of blood products [5]. 

Management Strategies

The two mainstay interventions for the management of saccular aneurysms include endovascular coiling and surgical clipping. Coiling is an endovascular technique whereby the aneurysm sac is densely packed, thereby inducing rapid coagulation that will isolate the aneurysm from active cerebral circulation [1]. Clipping is a microsurgical technique that involves placing a clip across the neck of the aneurysm while maintaining patency of the parent vessel. The decision-making process for which method to employ is complex, with providers taking into account both patient and aneurysm-specific factors. Patient factors may include symptomatic vs asymptomatic status, medical comorbidities, lifestyle risk factors such as smoking and alcohol use, patient age, and individual vascular anatomy. Aneurysm based factors may include size, location, neck width, and other morphological features [1,7]. Management approach will also vary depending on rupture status. 

Generally, for coiling, an unassisted approach may be used for aneurysms with favorable anatomical features such as narrow neck width and a favorable relationship with branching vessels [1]. Less anatomically favorable or more complex saccular aneurysms may require assisted coiling methods or flow-diverting stents [1]. Wide-neck saccular aneurysms, those with a neck-width greater than 4 mm or a dome-neck ratio less than 2, require special management considerations. These typically necessitate more complex clipping operations or the use of endovascular adjuncts such as stents or flow-diverters to avoid coil protrusion [7].

**Figure 1:**
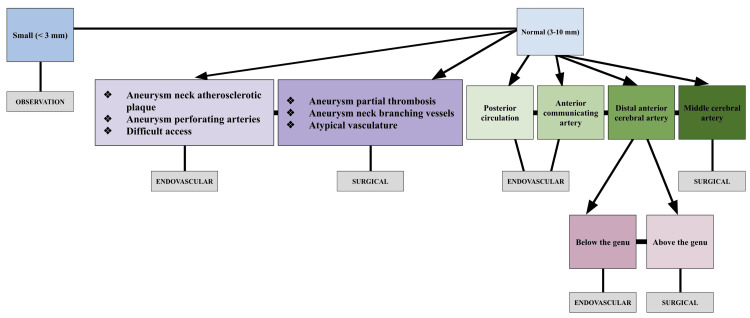
Management flowchart for unruptured saccular aneurysms. Blue: size; Purple: complexity; Green: location; Pink: location relative to the corpus collosum; Grey: management modality. mm: millimeters.

For small unruptured aneurysms, those less than 3 mm in diameter, current guidelines advise observation as only 13% of SAH are attributable to this subset [7]. Location also plays a role in management of unruptured aneurysms, with posterior circulation and AComm aneurysms typically treated endovascularly, and MCA aneurysms classically treated surgically [7]. Unruptured distal anterior cerebral artery (DACA) aneurysms are shown to be safe and effective candidates for both surgical and endovascular approaches, and providers commonly use more specific anatomical factors, such as location relative to the genu of the corpus callosum, to determine intervention modality [7]. Those that lie above the genu are preferentially treated surgically, while those that lie below the genu are treated endovascularly due to their accessibility [6]. Other anatomical considerations make patients with unruptured saccular aneurysms a better candidate for one approach over the other. The presence of a partial thrombosis, vessels branching from the aneurysm neck, and atypical extracranial vasculature make patients more suitable for surgical clipping, while atherosclerotic plaques at the aneurysm neck, perforating arteries, or difficult surgical access make patients more suitable for endovascular coiling. Figure 1 provides a flow diagram demonstrating surgical vs endovascular management of unruptured saccular aneurysms for the above-mentioned sizes, complexities, and locations. 

**Figure 2:**
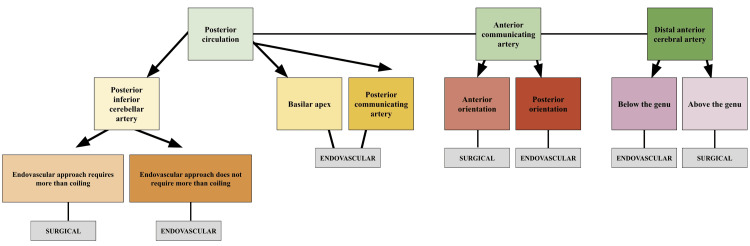
Management flowchart for ruptured saccular aneurysms. Green: location; Yellow: location within the posterior circulation; Orange: endovascular complexity; Red: orientation; Pink: location relative to the corpus collosum; Grey: management modality.

For the management of ruptured saccular aneurysms, large and giant aneurysms (>10 mm and >25 mm respectively) typically require adjunct endovascular methods such as stenting or flow-diversion, or complex clip reconstructions or bypass surgery [6]. Ruptured aneurysms will also often require a ventriculostomy in the case of hydrocephalus [6]. With increased size, where both clipping and coiling are difficult and high-risk interventions, morphology and location must be considered in conjunction [6]. In the setting of a wide-necked ruptured aneurysm, endovascular adjuncts such as stent-assisted coiling (SAC) or flow diverters may be employed to help divert flow away from the aneurysm’s neck. To avoid the use of dual antiplatelet therapy (DAPT) necessitated by these interventions, which increases ventricular drain-related bleeds from 9% to 20.9%, wide-necked ruptured saccular aneurysms may also be managed using intrasaccular flow disrupters such as Woven EndoBridge (WEB) [6]. Given that these devices avoid protrusion into the parent vessel, DAPT is not always required post-operatively, helping to mitigate bleeding risk [6]. With regards to location, posterior circulation aneurysms, including basilar apex, PICA, and PComm aneurysms are typically managed endovascularly. The reasoning for this is that the deeper location of these aneurysms, important perforators, and surrounding cranial nerves, bring a higher surgical risk. The exception to this is when the endovascular approach entails more than just coiling for PICA aneurysms, in which case clipping may be the preferred intervention [6]. For ruptured AComm aneurysms, orientation is an essential consideration for management approach, with those which are oriented anteriorly being more readily accessible via clipping [6]. Posteriorly oriented AComm aneurysms are typically coiled, as clipping has been associated with subsequent vessel ischemia [6]. As with unruptured saccular DACA aneurysms, those that are ruptured can be managed via either coiling or clipping, with many surgeons taking the relative position of the genu of the corpus callosum into consideration. For ruptured MCA aneurysms there is currently mixed consensus on optimal management approach. Figure 2 provides a flow diagram demonstrating surgical vs endovascular management of ruptured saccular aneurysms for the above-mentioned locations and complexities. 

*Prognosis* 

Patient outcomes vary based on the rupture status of the aneurysm and any number of the above discussed patient and aneurysmal factors. The majority of unruptured aneurysms discovered incidentally won't ever rupture or become problematic [5]. As mentioned previously, only 0.7% to 1.9% of patients with existing saccular aneurysms will experience a rupture. When aneurysm rupture occurs, prognosis is influenced by a myriad of factors including age, medical comorbidities, aneurysm location, Fisher grade, Hunt-Hess grade, and treatment success [5]. Among those with ruptured berry aneurysms, 25% of patients will die within the first 24 hours. Of those who survive past 24 hours, another 25% will pass away within the next six months from the development of complications [5]. Complications include rebleeding, hydrocephalus, vasospasm, seizures, cardiac stress, and hyponatremia which all influence overall prognosis [5]. 

Fusiform aneurysms

Definition and Epidemiology

Fusiform aneurysms are a subtype of non-saccular aneurysm characterized by circumferential dilation of an entire vessel wall for a short distance [8]. Those encompassing a relatively longer vessel distance are termed “cylindrical” [8]. Fusiform aneurysms are a rare subtype of aneurysm, accounting for between 3% and 13% of all intracranial aneurysms. They are most commonly seen in the vertebrobasilar system [8]. In a retrospective study by Park et al. encompassing 2,458 aneurysms, the mean age of patients at time of diagnosis was 45, with a male:female ratio of 1.4:1 [8]. This is consistent with numerous other studies previously conducted, supporting the notion that spontaneous fusiform aneurysms are more common in men and are typically found in younger patients [8]. 

Pathophysiology

The pathophysiology of fusiform aneurysms is most commonly secondary to atherosclerosis or vessel dissection [8]. An essential feature of these aneurysms is the communication between the true lumen and the pseudo-lumen via a disruption in the internal elastic lamina [9]. The initial inciting event is lipid accumulation within and beneath the intima in the case of atherosclerosis, or dissection of the internal elastic lamina in the case of vessel dissection. This then results in disruption of the internal elastic membrane and invasion of the muscular wall with resulting intramural hemorrhage or formation of an intramural hematoma respectively [9]. This is followed by transmural expansion of the thrombus which thickens the intima to create a fusiform shaped aneurysm [9]. From here there are five main evolution patterns including further expansion of the intramural hematoma, enlargement of the hematoma with resulting circumferential vessel enlargement, the formation of serpentine channels, aneurysm rupture outward, or aneurysm rupture into the arterial lumen [9]. This may then progress to SAH or ischemia depending on the location of the dissection caused by the resulting hematoma from bleeding into the arterial wall [9]. Their etiology may less commonly be attributed to disorders of collagen or elastin, infection, or very rarely by a neoplastic invasion of the vessel wall [8]. Each of these causes weakening of the arterial wall and allows for aneurysm formation, with a similar sequela of events as described above. 

Clinical Features

These aneurysms may present in a variety of ways depending on morphology, ranging from asymptomatic to transient ischemic attack (TIA) or stroke to hemorrhage. Patients may demonstrate symptoms of vessel occlusion presenting as ischemic stroke of the brainstem, arterial rupture, or mass effect from compression of the brainstem, cerebellum, or cranial nerves [9,10]. Most commonly, they will present as an ischemic stroke or as mass effect [10]. This may present with symptoms including but not limited to aphasia, ataxia, diplopia, dizziness, cranial nerve deficit, and hemiparesis [9]. If the tunica adventitia is disrupted, then the aneurysm may rupture and progress to a SAH [9]. Alternatively, if the aneurysm remains contained by the tunica media, there will be enlargement of the aneurysm towards the vessel lumen, with ischemia or stenosis of the artery ensuing [9]. 

Diagnosis 

As with other types of aneurysms, non-invasive diagnostic imaging is typically first employed with CTA and/or MRA. However, DSA remains the gold standard. The shape of these aneurysms helps clinicians differentiate them from saccular aneurysms, as they will demonstrate circumferential as opposed to one-sided ballooning of the vessel wall [9]. Fleming’s classification system can be used to differentiate non-saccular vertebrobasilar circulation aneurysms, such as fusiform, based on their radiographic appearance [11]. Generally, the presence of the non-saccular aneurysm is defined when dilation of the vessel is greater than 1.5 times the normal diameter and there is absence of an aneurysm neck [11]. From there the aneurysm may be categorized as (1) fusiform: aneurysmal dilation involving a portion of the vessel with no clearly defined neck, (2) dolichoectasia: uniform dilation of the entire vessel and associated tortuosity, (3) transitional: uniform dilation of the entire vessel with superimposed dilation of a portion of the arterial segment, or (4) indeterminate [11]. 

Management

**Figure 3:**
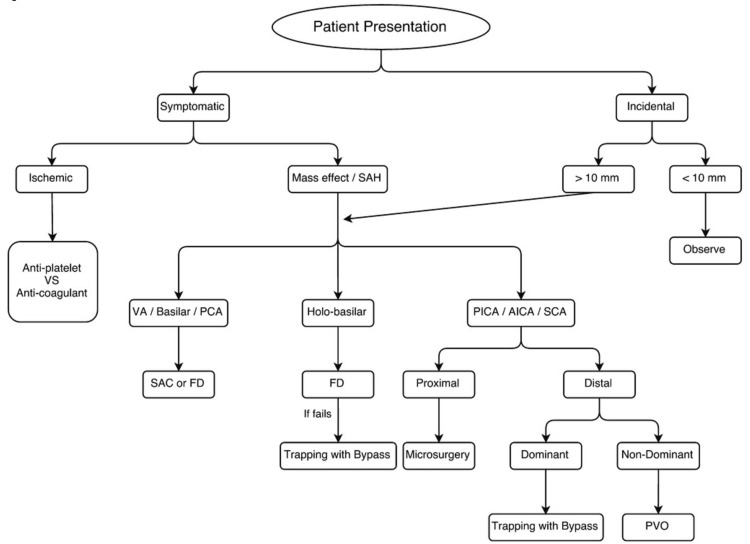
Journal of Neurosurgery suggested treatment algorithm for fusiform aneurysms in the posterior circulation based on presentation. FD: flow diverter; PVO: parent vessel occlusion; SAC: stent assisted coiling. [11] This figure is protected by Copyright, is owned by Journal of Neurosurgery Publishing Group (JNSPG), and is used with permission only within this document. Permission to use it otherwise must be secured from JNSPG. Full text of the article containing the original figure is available at thejns.org.

Nearly all ruptured fusiform aneurysms will require invasive intervention. Unruptured fusiform aneurysms greater than 10 mm in diameter are also an indication for intervention [11]. Figure 3 provides a diagram of the *Journal of Neurosurgery’s* suggested treatment algorithm based on patient presentation and various aneurysmal characteristics [11]. 

Management of fusiform aneurysms is more complicated as compared to their saccular counterparts, as they lack a true aneurysm neck, thus rendering clip reconstruction and coil embolization nearly impossible. Interventions instead require more advanced microsurgical techniques to reduce or reverse blood flow, or newer interventions such as endovascular flow diversion [11]. Microsurgical technique via clipping is typically reserved for aneurysms which cannot be managed endovascularly. If employed, flow reduction or bypass/trapping is used in the setting of poor collateral blood supply, flow reversal is used in the setting of sufficient collateral supply, and trapping may be used to decompress aneurysms with associated mass effect [11]. Management over the last decade has evolved to endovascular techniques with the use of SAC or flow diversion instead, which has shown to have acceptable outcomes in the majority of patients [11]. Patients undergoing this intervention will require post-operative DAPT for a minimum of three to six months, followed by aspirin for life [11]. 

*Prognosis* 

Prognosis of fusiform aneurysms varies depending on ruptured vs unruptured status and characteristics of the aneurysm. For unruptured aneurysms, those less than 7 mm in maximum diameter and not associated with atherosclerosis have a low risk of rupture or becoming symptomatic for several years after discovery [12]. Those that are greater than 7 mm in maximum diameter or are symptomatic have an increased risk of enlargement and possible rupture [12]. Unruptured fusiform aneurysms with associated atherosclerosis have a worse prognosis given their challenging management [12]. For patients with ruptured fusiform aneurysms, rebleeding rates range from 30% to 85% [11]. When ruptured fusiform aneurysms are managed conservatively (without surgical intervention), mortality rate is around 38% over a follow-up period of 18 months [11]. 

Mycotic aneurysms 

Definition and Epidemiology

A mycotic aneurysm is a dilation in a blood vessel wall that develops in response to an infectious etiology, secondary to infective endocarditis (IE) from a bacterial pathogen in nearly 90% of cases [13,14]. The term “mycotic” was created by William Osler as he described the fungal appearance of multiple aneurysms in a man with valve vegetations [13]. The incidence of such aneurysms is fairly rare, accounting for 0.7% to 3% of all cerebral aneurysms [13]. They are more commonly found in men than women, presumably due to higher rates of atherosclerosis, smoking, and diabetes, which are all associated risk factors [13]. The median age of affected patients is 65 years [13]. Common infectious pathogens include *Staph. aureus* and *Strep. viridians* [14]. Fungal or viral pathogens may be implicated in immunocompromised patients. In patients with IE, 2% to 10% of patients will develop cerebral mycotic aneurysms, more commonly seen with left-sided IE [13].

Pathophysiology

The etiology of mycotic aneurysms is secondary to either local infectious invasion of a vessel wall in a patient with a pre-existing aneurysm or atheromatous plaque, or in the setting of systemic infection with bacteremia. The bacterial invasion of the blood vessel weakens the arterial structure and leads to a resulting dilatation [13]. Once the infection seeds the vessel intima, the deeper layers of the vessel are rapidly degraded as well. Additionally, the vessel infection stimulates recruitment of pro-inflammatory cytokines which attract neutrophils that subsequently cause further breakdown of the vessel wall via activation of matrix metalloproteinases (MMP) [13]. 

There are multiple factors implicated in the pathogenesis of mycotic aneurysms, including bacteremia, local injury, local spread, and septic emboli. In patients with pre-existing aneurysms or atherosclerosis, such as the elderly, bacterial seeding in the setting of sepsis or bacteremia is facilitated by existing vessel injury [13]. Local injury to a blood vessel wall via means such as intravenous drug use, trauma, or iatrogenic causes allows for invasion of bacteria into the vessel wall and resulting aneurysm formation [13]. Local spread may result from intracerebral pathologies such as meningitis, abscess, cavernous sinus thrombophlebitis, or orbital cellulitis [14]. For cerebral mycotic aneurysms, the most commonly implicated etiology is septic emboli from IE, accounting for 90% of cases [14]. These typically result in multi-focal dilations (present in up to 25% of patients) and are commonly located at peripheral branch points within the cerebral vasculature if of bacterial origin [13,14]. When located peripherally, the MCA is commonly implicated (specifically the M2 segment and beyond) [14]. If of fungal origin, aneurysms are more commonly located in long proximal segments of the Circle of Willis [14]. Based on these etiologies, patients who are intravenous drug users, or those with immune compromised states, are at a higher risk for aneurysm development.

Clinical Features

A patient’s clinical presentation will vary depending on the site of the aneurysm, the severity of the infection, and patient comorbidities [13]. Common initial presentation for unruptured aneurysms includes fever, weight loss, and headache [13,14]. Given the relatively non-specific nature of this presentation, many patients remain undiagnosed until the aneurysm progresses and causes more severe symptoms. The natural progression may include expansion, pseudoaneurysm formation, rupture, hemorrhage, sepsis, and multiorgan failure [13]. After progression, patients may present with neurologic deficits, stroke, or SAH [13]. In patients who present with a ruptured mycotic aneurysm, they will exhibit symptoms of intracranial hemorrhage [14]. 

Diagnosis 

A combination of clinical, laboratory, imaging, and intraoperative findings are used for diagnosis. Clinical findings include the above-mentioned symptoms, in combination with known patient risk factors. General laboratory findings will indicate the presence of an infection [13]. Blood cultures are also typically used to aid in diagnosis, with a positive culture in 50% to 85% of cases [13]. As mentioned, commonly implicated bacterial organisms include *Staph. aureus* and *Strep. viridians*, accounting for up to 90% of cases. While fairly rare, fungal or viral organisms may be responsible in immunocompromised patients such as those with diabetes, HIV, or those undergoing chemotherapy [13]. Imaging findings using head CT with contrast, MRA with contrast, and DSA are the most sensitive and specific diagnostic tool for these aneurysms. On initial examination of imaging findings, mycotic aneurysms can often be differentiated from saccular aneurysms based on their typically peripheral location within the vasculature [14]. Highly suggestive imaging findings include a saccular lesion with lobulated contours, soft tissue inflammation around the vessel wall, air collection surrounding or within the vessel wall, or fluid collection around the aneurysm [13]. If operative samples of the aneurysms are taken and sent to pathology, transmural inflammation with abscesses, necrosis, and thrombosis will be present in the tissue [13]. 

Management

**Figure 4:**
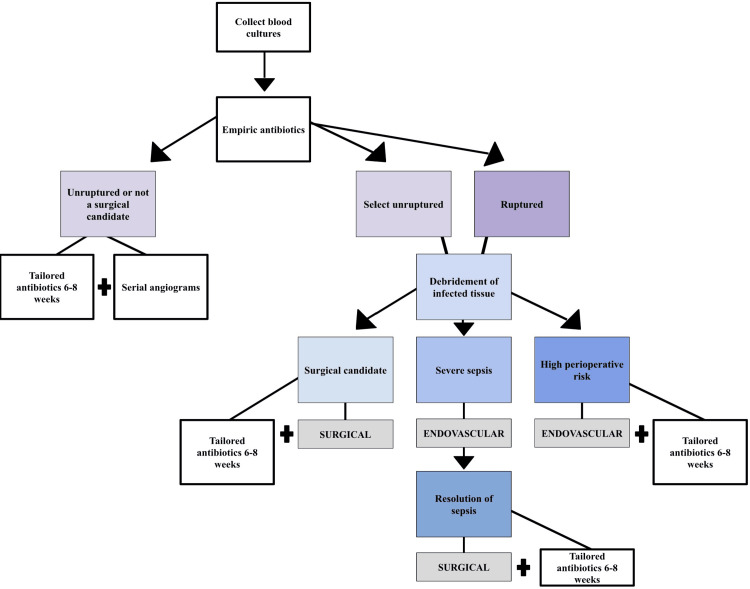
Management flowchart for mycotic aneurysms. Purple: rupture status; Blue: surgical steps and details; Grey: surgical vs endovascular management modality.

Given its infectious etiology, all patients require antibiotic therapy. While awaiting blood culture results, patients are typically started on an empiric regimen of vancomycin with ceftriaxone or piperacillin-tazobactam for broad spectrum coverage. Once the pathogen and sensitivities result, patients will receive tailored antibiotics for a baseline duration of six to eight weeks [13]. Patient therapeutic response is continually monitored via laboratory studies and patient clinical status [13]. While surgical resection is the definitive management for mycotic aneurysms, patients with unruptured aneurysms or those who are not suitable surgical candidates may receive conservative management with antibiotics alone [14]. When implemented in suitable candidates, the conservative approach results in the complete resolution of approximately 30% of aneurysms, decreased size in 20%, no significant change in size in 15% to 30%, and an increase in size in 20% of patients [14]. This approach requires serial angiographic follow-up for aneurysm monitoring [14]. Surgical intervention in conjunction with antibiotic therapy may be implemented for either unruptured or ruptured aneurysms. This entails debridement of all infected tissue with subsequent revascularization through either an open (clipping) or endovascular (coiling) approach [13]. Endovascular technique is shown to be a suitable option for patients with a high perioperative risk or as a temporary bridging intervention for those with severe sepsis [13]. Figure 4 provides a flow diagram demonstrating management of mycotic aneurysms for the above-mentioned presentations.

Prognosis 

Even for patients who undergo appropriate management, prognosis is poor [14]. Unruptured aneurysms have an overall mortality rate of up to 30%, and their ruptured counterparts up to 80%. Mycotic aneurysms have a very high perioperative mortality rate [13]. Perioperative mortality can be attributed to prolonged operating times with resulting distant ischemia causing a cerebrovascular accident [13]. For patients who undergo open surgery, the perioperative mortality rate is roughly 20%, thus increasing interest in employing endovascular techniques for management [13]. It is worth noting however that the long-term outcomes for patients who survive surgical intervention are comparable [13]. Post-operatively, patients with grafts placed may suffer from reinfection or graft failure, with infections having a 100% mortality rate within two years if the implicated graft is not removed [13]. Post-operative complications may occur in up to 60% of patients. Patients who go untreated have a rupture risk of approximately 60%, and are also at risk for complications including sepsis, thrombosis, stroke, and multiorgan failure [13]. As discussed previously, MMP are implicated in the pathogenesis of mycotic aneurysms through their role in vessel breakdown. Given their key role, higher levels in patients may be used as a prognostic indicator for risk of rupture [13]. Additionally, patients with multiple intracerebral mycotic aneurysms have a poorer prognosis [14]. 

Marantic (non-bacterial thrombotic) aneurysms

Definition and Epidemiology

Marantic aneurysms are associated with non-bacterial thrombotic endocarditis (NBTE) or systemic conditions such as cancer and autoimmune diseases. The hypercoagulability of these diseases contributes to sterile thrombus formation that leads to aneurysm development. The incidence of these aneurysms is extremely rare, making their diagnosis and management a challenge [15]. 

Pathophysiology

The pathogenesis of marantic aneurysms involves vessel wall weakening secondary to systemic factors, including malignancy or autoimmune disorders. One suggestion of how malignancies cause aneurysms is that tumor embolus invades the vessels and causes dilatation, leading to an aneurysm formation [15]. In both malignancy and autoimmune disorders, endothelial injury promotes thrombus formation along arterial walls [16]. Over time, these thrombi contribute to vascular dilation leading to aneurysm formation. 

Clinical Features 

Marantic aneurysms are often undetected until complications such as rupture occur. Depending on the type of disease and neoplasm, the chance of rupture varies. For example, all choriocarcinoma aneurysms presented with SAH, while only 19.6% of the cerebral marantic aneurysms secondary to cardiac myxoma presented with SAH [15].

Diagnosis and Imaging 

Imaging studies play a critical role in the diagnosis of marantic aneurysms. Similar to other types of aneurysms, CTA or DSA can be used to visualize the aneurysm [17]. Coagulation studies, autoimmune markers, cancer markers, and, in select cases, biopsy can aid in identifying underlying systemic conditions and ruling out infectious causes [17]. 

Management 

Marantic aneurysms are such a rare type of cerebral aneurysm that a definitive treatment has yet to be established. Possible treatment options include addressing the underlying systemic disease to reduce the hypercoagulable state, and anticoagulation therapy, using agents such as warfarin or direct oral anticoagulants (DOACs). Invasive surgical interventions such as aneurysm clipping or stenting can be considered in cases of large or symptomatic aneurysms [17]. 

Prognosis

The prognosis of marantic aneurysms depends heavily on the underlying systemic disease, as well as the size and location of the aneurysm. Complications, including rupture, embolism, and ischemia, significantly impact outcomes. Prognosis is often poor in patients with malignancy, whereas those with autoimmune or hypercoagulable conditions tend to have a better outlook when both the aneurysm and the systemic condition are adequately treated [15]. 

Blister aneurysms

Definition and Epidemiology

Blister aneurysms are small, fragile lesions that typically form at non-branching points of major intracranial arteries. These aneurysms often appear as sidewall lesions with hemispheric or broad-based morphologies, lacking an identifiable neck [18]. They are most commonly found in the supraclinoid segment of the internal carotid artery. They go through rapid morphological changes on angiographic follow-up, and their high fragility makes them prone to rupture or regrowth. Blister aneurysms account for 0.3% to 1% of all intracranial aneurysms and 6.6% of ruptured cases [19]. 

Pathophysiology

The etiology of blister aneurysms is poorly understood, but several mechanisms have been proposed. These lesions may result from defects in the arterial wall combined with hemodynamic stress, with ruptured cases often linked to underlying internal carotid artery dissection [20]. Surgical observations have revealed proximal intramural hematomas in some cases, suggesting a pathophysiological mechanism involving retrograde formation and progression of these lesions. Other potential contributors include mechanical stress, acute inflammation, increased cell apoptosis, and elevated levels of cellular proliferation [20].

*Clinical Features* 

Preexisting intracranial atherosclerosis and elevated wall shear stress (WSS) or wall shear stress gradients (WSSG) may predispose individuals to blister aneurysm development [20]. Unique morphological features, such as a platelet plug overlying thin adventitia that lacks a collagen layer, are thought to contribute to their propensity for rapid size and shape changes and high rupture risk [20].

Diagnosis

Blister aneurysms are challenging to diagnose due to their small size, unusual morphology, and atypical locations. Patients often present with SAH, rapidly evolving clinical courses, and imaging findings that reveal small, broad-based irregularities in the vessel wall. Noninvasive imaging modalities, such as CTA and MRA, may fail to detect these lesions due to adjacent bony structures and atypical locations [21]. DSA remains the gold standard for diagnosis, often revealing features such as wide necks, diverse shapes, and parent artery involvement near the aneurysmal neck. Changes in morphology over short periods and stasis of contrast within the aneurysm are also characteristic findings [21]. 

Management 

Treatment of blister aneurysms is challenging due to their high fragility and susceptibility to rupture during intervention. Surgical options include wrapping, clipping, suturing, and trapping with or without bypass procedures, but these approaches are associated with significant risks of intraoperative rupture and postoperative rebleeding [21]. Endovascular strategies, such as coiling, stenting, or the use of flow-diverting stents, have gained favor due to lower morbidity and mortality rates when compared to open surgery [18]. Flow-diverting stents have shown promise with fewer perioperative complications and comparable functional outcomes. However, long-term outcomes remain uncertain, and no single treatment modality has been established as superior in the literature [18]. 

*Prognosis* 

Blister aneurysms carry a high risk of complications, with significant morbidity and mortality rates if not managed promptly and effectively. Their extreme fragility, adjacent parent artery involvement, and tendency to rupture contribute to their poor prognosis. Early intervention is critical to prevent rebleeding [21]. Outcomes are highly dependent on early diagnosis, appropriate management, and the ability to mitigate risks during treatment.

Dissecting aneurysms

Definition and Epidemiology

Dissecting aneurysms result from arterial wall dissection, which leads to the formation of pseudoaneurysms. Cranio-cervical dissections are implicated in 15% to 20% of strokes among young individuals [22]. These aneurysms often occur in the carotid or vertebral arteries and can be bilateral in up to 15% of cases [23]. The most common sites include the MCA (41%), vertebrobasilar circulation (23%), internal carotid artery (21%), and ACA (13%) [23]. Individuals with connective tissue disorders, such as Marfan syndrome, Ehlers-Danlos syndrome, neurofibromatosis type 1 (NF1), and Loeys-Dietz syndrome, are at an elevated risk due to structural abnormalities in the extracellular matrix, including defects in collagen and proteoglycans, which weaken the vessel walls [24]. 

Pathophysiology

Dissecting aneurysms may result from trauma or underlying connective tissue disorders. Intraluminal blood penetrating the vessel wall creates an intramural hematoma, which, depending on its extension, may cause ischemia, embolization, or a dissecting aneurysm [23]. The dissection begins between the intima and media layers of the vessel wall, leading to the destruction of all layers. Progression of the dissection and subsequent healing can result in large, partially thrombosed aneurysms and recurrent episodes of dissection. 

*Clinical Features* 

Symptoms vary based on the site and extent of the dissection. Common presentations include headache, neurological deficits, and ischemic symptoms due to vessel occlusion or embolization. Rarely, bleeding into adjacent structures, cranial nerve paresis, or Horner’s syndrome may occur. Intradural internal carotid artery dissection presents with unilateral headache followed by stroke symptoms; less commonly, SAH. MCA and ACA dissections present with stroke symptoms. Intradural vertebral artery dissection typically presents with SAH, headache, vertigo, nausea, vomiting, and lateral medullary syndrome [24]. 

*Diagnosis* 

DSA is the gold standard for diagnosing dissections. Key angiographic findings include contrast stagnation in the aneurysmal pouch, stenotic segments proximal or distal to the ectasia, and a fusiform appearance [23]. Other diagnostic signs include the "pearl and string" sign and intramural hematomas visible as thick, ring-like enhancement on contrast imaging or double-lumen signs. T1-weighted MRI can detect intramural hematomas as areas of increased signal intensity. 

*Management* 

Management of dissecting aneurysms depends on the severity of symptoms and the location of the dissection. Most stenosis caused by extradural dissections resolve with anticoagulation therapy (e.g., heparin). However, surgical or endovascular treatment is indicated for persistent ischemic symptoms or cases with a high risk of rupture. Options include clipping, wrapping, or bypass procedures for diseased segments and coiling, stenting, or balloon dilatation for acute or recurrent strokes unresponsive to pharmacological treatment [23].

*Prognosis* 

The prognosis of dissecting aneurysms varies based on the dissection’s location and the chosen treatment approach. Intradural dissections associated with SAH carry a high risk of morbidity and mortality, especially after rebleeding [25]. Aggressive surgical or endovascular treatment is often necessary to prevent complications. Patients with acute or recurrent strokes unresponsive to medical therapy benefit from stenting, balloon dilatation, or bypass procedures to restore vascular integrity [25]. Prompt intervention is critical to improving outcomes and minimizing the risks of severe complications such as ischemia, cranial nerve palsies, and neurological deficits.

Table 1 below synthesizes all of the above information on pathophysiology, clinical presentation, imaging, risk of rupture, and treatment based on each aneurysm type. 

**Table 1:** Comparative table on different aneurysm types. CT: computerized tomography; MRI: magnetic resonance imaging; CTA: computerized tomography angiography; DSA: digital subtraction angiography; SAH: subarachnoid hemorrhage; ICA: internal carotid artery.

Aneurysm type	Pathophysiology	Presentation	Imaging	Risk of rupture	Treatment
Saccular	Result from weakness at arterial branch points due to factors like hypertension and genetic predispositions. Hemodynamic stress plays a role.	Often asymptomatic unless ruptured. Symptoms of rupture include "thunderclap headache," meningeal signs, and neurological deficits.	Diagnosed using CT, MRI, CTA, or DSA. Saccular morphology at arterial branch points.	High in posterior circulation, large size, or irregular shapes	Endovascular coiling for most cases; surgical clipping for accessible or wide-neck aneurysms. Posterior circulation often requires endovascular methods.
Fusiform	Circumferential vessel dilation often secondary to atherosclerosis or vessel dissection. Rare and found in vertebrobasilar regions.	Symptoms of mass effect or ischemia; less commonly hemorrhage.	No clear neck, circumferential dilation. DSA is critical for diagnosis.	Larger (>7 mm) aneurysms or symptomatic ones carry higher rupture risks.	Endovascular flow diversion or stenting preferred. Surgical trapping or bypass for complex cases.
Mycotic	Arise from infection, commonly bacterial. Vessel wall weakened by septic emboli or direct invasion.	Fever, weight loss, headache initially. Rupture leads to SAH or focal neurological deficits.	Peripheral lesions with inflammation; CTA and DSA are diagnostic.	High if untreated; linked to systemic infection severity.	Antibiotics with possible surgical debridement or endovascular intervention.
Marantic	Associated with malignancy or autoimmune diseases. Hypercoagulable states lead to thrombus-induced aneurysms.	Often asymptomatic until rupture.	CTA and DSA used; findings depend on underlying disease.	Higher with certain malignancies (e.g., choriocarcinoma).	Address systemic conditions with anticoagulation. Surgery reserved for symptomatic aneurysms.
Blister	Fragile lesions without a neck, often linked to arterial dissection or mechanical stress. Found in the supraclinoid ICA.	Commonly presents with SAH due to rupture.	Challenging to identify due to small size and unusual morphology. DSA is gold standard.	Extremely high due to fragility.	Flow-diverting stents preferred. Surgical options carry high risk of intraoperative rupture.
Dissecting	Arise from arterial dissection with intramural hematoma formation. Common in carotid or vertebral arteries.	Symptoms of ischemia or SAH depending on location.	Pearl and string sign, double-lumen on DSA or MRI.	High for intradural aneurysms; requires prompt intervention.	Anticoagulation for ischemic symptoms; stenting, coiling, or bypass for ruptures or high-risk lesions.

Future directions

The field of intracranial aneurysm management continues to evolve with ongoing advancements in imaging, treatment modalities, and understanding of underlying etiological factors. Future research directions focus on improving diagnostic precision, optimizing treatment strategies, and developing preventative measures.

The differentiation of various aneurysm types remains a significant challenge due to overlapping imaging features, particularly for rarer forms such as blister, mycotic, and marantic aneurysms. Emerging imaging technologies, including high-resolution vessel wall imaging (HR-VWI) with MRI, hold promise for visualizing aneurysm wall composition, inflammation, and stability [26]. HR-VWI can evaluate vessel wall and detect pathological changes that could be hard to identify on conventional imaging [26]. Such advancements could aid in identifying aneurysms at higher rupture risk and distinguishing between infectious or noninfectious etiologies. Similarly, the application of artificial intelligence (AI) and machine learning algorithms in imaging analysis has the potential to improve detection accuracy, predict rupture risk, and guide treatment planning [27]. These innovations may allow for earlier identification of aneurysms, particularly those with atypical or subtle presentations, ultimately enhancing patient outcomes.

Treatment modalities for intracranial aneurysms continue to advance, particularly in endovascular techniques. Flow-diverting stents, which have already revolutionized the management of complex aneurysms, are being refined to improve safety and efficacy, particularly for small, distal, and wide-necked aneurysms. Innovations such as bioresorbable scaffolds and advanced coatings to reduce thrombogenicity and inflammation are under investigation [28,29]. For surgical treatments, the development of robotic-assisted microsurgery offers the potential to improve precision in complex cases while minimizing complications. These technologies could expand the scope of surgical clipping, particularly for aneurysms in challenging locations.

Further research is needed to understand the underlying genetic and molecular causes of aneurysm formation and progression. Studies exploring the role of specific genetic mutations, epigenetic modifications, and biomarkers could help identify individuals at higher risk for aneurysm development or rupture, paving the way for targeted surveillance and preventive strategies.

Additionally, the exploration of preventative approaches, such as lifestyle modifications, pharmacological interventions (e.g., antihypertensive and antiplatelet therapies), and novel vascular protective agents, is critical. Trials assessing the long-term efficacy of such strategies in high-risk populations could transform current management standards.

Lastly, longitudinal studies to better define the natural history of unruptured aneurysms are necessary to refine risk stratification tools and improve clinical decision-making. Expanding data on aneurysms in specific populations, such as pediatric patients and individuals with connective tissue disorders, will also enhance personalized management approaches.

## Conclusions

Intracranial aneurysms represent a diverse group of cerebrovascular pathologies, each with unique epidemiology, pathophysiology, clinical features, and treatment considerations. Saccular aneurysms are the most common type, while rarer forms such as fusiform, mycotic, marantic, blister, and dissecting aneurysms require specialized approaches to diagnosis and management. This review highlights the distinct characteristics and challenges associated with each type, emphasizing the importance of understanding their unique features to optimize patient outcomes. Early identification of aneurysms, whether asymptomatic or symptomatic, is crucial for preventing catastrophic complications such as rupture. Advances in imaging modalities have significantly improved the ability to detect and characterize aneurysms, but further development in techniques such as high-resolution vessel wall imaging and artificial intelligence-based analysis is needed to enhance diagnostic precision. Tailored management strategies, ranging from endovascular interventions to surgical techniques, are critical to addressing the anatomical and physiological complexities of different aneurysm types. Incorporating patient-specific factors into decision-making ensures optimal outcomes and minimizes treatment-related risks. By addressing existing knowledge gaps and leveraging technological breakthroughs, the field can move closer to precise diagnostics, safer interventions, and effective preventative measures for this life-threatening condition.

In conclusion, the management of intracranial aneurysms requires a multidisciplinary approach that integrates cutting-edge technology, personalized care, and ongoing research. By advancing our knowledge and tools, we can improve the prognosis and quality of life for patients with these complex and life-threatening conditions.
